# Multiple Applications of Different Exercise Modalities with Rodents

**DOI:** 10.1155/2021/3898710

**Published:** 2021-11-25

**Authors:** Denise Börzsei, Renáta Szabó, Alexandra Hoffmann, Attila Harmath, Judith Sebestyén, Jasmin Osman, Béla Juhász, Dániel Priksz, Csaba Varga, Anikó Pósa

**Affiliations:** ^1^Department of Physiology, Anatomy and Neuroscience, Faculty of Science and Informatics, University of Szeged, Szeged 6726, Hungary; ^2^Department of Physiology, Anatomy and Neuroscience, Interdisciplinary Excellence Centre, University of Szeged, Szeged 6726, Hungary; ^3^HR-Pharma Ltd., Szeged 6726, Hungary; ^4^KEL-FEDER PLC., Tompa 6422, Hungary; ^5^Department of Burns and Plastic Surgery, South-Pest Hospital Centre, National Institute for Infectology and Haematology, Budapest 1097, Hungary; ^6^Department of Pharmacology and Pharmacotherapy, Faculty of Medicine, University of Debrecen, Debrecen 4032, Hungary

## Abstract

A large proportion of chronic diseases can be derived from a sedentary lifestyle. Raising physical activity awareness is indispensable, as lack of exercise is the fourth most common cause of death worldwide. Animal models in different research fields serve as important tools in the study of acute or chronic noncommunicable disorders. With the help of animal-based exercise research, exercise-mediated complex antioxidant and inflammatory pathways can be explored, which knowledge can be transferred to human studies. Whereas sustained physical activity has an enormous number of beneficial effects on many organ systems, these animal models are easily applicable in several research areas. This review is aimed at providing an overall picture of scientific research studies using animal models with a focus on different training modalities. Without wishing to be exhaustive, the most commonly used forms of exercise are presented.

## 1. Introduction

Sport can be thought of as a therapeutic tool or a prevention strategy for different disorders. It is beginning to be learned that physical exercise exerts its effects via extensive molecular pathways by which it maintains and improves the quality of life. It is well known that regularly maintained training has several beneficial effects on overall health, from cells to whole organ systems [1]. Nonetheless, physical inactivity entails numerous health issues from systemic inflammation to hormonal dysfunctions which raises the risk of wide-ranging noncommunicable diseases, such as type II diabetes, metabolic syndrome, cardiovascular and neurodegenerative disorders, and even cancer [2–5]. Over and above, sedentary lifestyle-related redox disturbance further aggravates preexisting pathological processes [6]. The main purpose of training-related research studies has been targeted on the health benefits of exercise to be able to prevent and treat these conditions. Comprehending the underpinning systemic changes provoked by exercise helps us to develop more efficient treatment methods and prevention strategies against widespread diseases. With the help of animal models, it is possible to study the most complex effects of exercise at all levels of organization. The appropriate animal species and the duration, frequency, and intensity of the training should be chosen according to the purpose of the study [7]. The most common types of exercise used in animal experiments are voluntary wheel running, forced wheel running, treadmill running, swimming, and resistance training [8, 9]. Aerobic exercise modalities are suitable for almost every noncommunicable disorder-related research area, while anaerobic training modalities are applicable in a much narrower field of research, including muscle formation studies [8–11]. In this review, we discuss the far-reaching benefits of physical exercise and its interpretation in different animal models. Our aim is to provide a comprehensive picture of the different exercise modalities used with rodents and their far-reaching effects on organ systems affecting the most researched noncommunicable diseases.

## 2. Beneficial Effects of Exercise on Different Organ Systems

### 2.1. Bones and Skeletal Muscle

Exercise has major effects on body composition. It is well established that physical activity improves bone properties such as bone quality or density. Consequently, it lowers the risk of osteoporosis. Osteoporosis is a condition characterized by low bone mass and bone fragility and mainly occurs among elderly people and postmenopausal women as a result of hormonal changes [12]. Exercise is considered to be the best nonpharmacologic approach in preventing osteoporosis; recent studies discussed that long-term exercise is able to increase bone strength and formation; therefore, it is effective in improving bone quality [13, 14].

Along with bones, exercise is able to increase muscle strength and improve balance and coordination. The most noticeable effect of long-term exercise, especially resistance training, is the increase of muscle mass. This process affects the basal metabolic rate and body composition in a favorable way. Besides, physical activity is proved to be promising in the regeneration and rejuvenation of muscle stem cells [15]. Exercise is also effective in age-related muscle atrophy, called sarcopenia. A recent work of White et al. supported the fact that long-term voluntary exercise can prevent sarcopenia in the hindlimb muscles in female and male rats as well [16]. Taken together, these results indicate that regular exercise has many beneficial effects on skeletal muscle function, regeneration, and bone quality at any age.

### 2.2. Metabolic Health

In the absence of exercise as a result of chronic positive energy balance, weight gain occurs. In this pathological condition, an increase in the number and size of adipocytes is observed, which leads to the disruption of leptin signaling and eventually to chronic inflammation [17]. On the contrary, exercise promotes metabolic health by decreasing body weight along with the amount of circulating lipids and the concentration of leptin and positively affects glucose tolerance and insulin sensitivity [18, 19]. Studies have shown that regular exercise significantly improved glucose homeostasis in diabetic and prediabetic status [20]. Moreover, regular exercise is efficient enough to reduce plasma leptin and insulin levels in hormone deficiency as well; thus, it plays an important part in the improvement of pathophysiological changes in connection with metabolic syndrome [21, 22]. Furthermore, exercise is able to increase the expression of glucose transporter 4 and contributed to balanced glucose homeostasis and insulin sensitivity in rats [23]. Hence, regular exercise is able to reduce the risk for metabolic disorders and the resulting cardiovascular complications.

### 2.3. Cardiovascular System

Physical exercise has far-reaching cardiovascular effects as well. Studies have shown that sustained physical activity lowers the individual's resting heart rate and blood pressure while it increases physiological cardiac hypertrophy [24, 25]. Exercise affects the cardiovascular system in different ways; it modulates numerous signaling pathways and improves oxygen delivery throughout the body via angiogenesis and vasodilation [26]. Nitrogen monoxide (NO) production in the endothelium rises significantly as a result of training and causes a well-known vasodilating phenomenon [27]. By enhancing nitrogen monoxide synthase activity, exercise has an undeniable role in the maintenance of normal blood pressure and in the treatment of hypertension [28]. Besides vasodilatation, NO has anti-inflammatory and platelet inhibitory effects as well, thereby contributing to the mitigation of atherosclerotic risk [29]. Additionally, exercise influences blood vessel morphology by extending the capillary network in the cardiovascular system. In a recent study of ours, we proved that a 12-week-long voluntary exercise was an effective therapeutic tool to improve cardiac function in aged rats; we clarified the exercise-moderated genetic modifications that contributed to the functional improvement of the heart [30]. Exercise can serve as a therapeutic tool after myocardial infarction (MI) as well; recent studies supported that after a postmyocardial injury, recreational exercise was able to improve cardiac health and antioxidant status [31, 32].

### 2.4. Nervous System

Numerous studies demonstrated the effects of physical activity on mental health, cognitive processes, and brain activity [33–35]. It is clarified that exercise affects several complex mechanisms including cerebral perfusion, neurogenesis, and synaptic plasticity [36, 37]. The findings of Kleemeyer et al. discussed that 6 months of exercise is associated with a hippocampal neuron density increase [38], while the work of Ruscheweyh et al. discovered a significant augmentation of the gray and white matter as a result of aerobic training [39]. Interestingly, encouraging results were obtained not only in young rats but also in older animals (19-month-old rodents). Scientists found that 1.5 months of voluntary exercise elevated gliogenesis by 20% and reverted age-related decline in neurogenesis by 50% [40]. Trophic factors such as the brain-derived trophic factor (BDNF) and vascular endothelial growth factor (VEGF) greatly support the cognition in young and older individuals as well. Several studies verified that running exercise at any age increased the expression of BDNF and VEGF in the hippocampus, which the phenomenon was correlated with the improvement of spatial learning and memory [41, 42]. In addition, exercise has been recommended as one of the best lifestyle interventions against neurodegenerative diseases (e.g., Alzheimer's disease, Parkinson's disease) [43]. It is well established that exercise has a crucial role in the protection of neurons, but recent results have also suggested that it is promising in the prevention of amyloid-*β* and tau protein plaque formation [44, 45]. According to recent studies, swimming, voluntary wheel running, and treadmill exercise have also proven to treat neuropathic pain in a mouse model [46, 47]. In addition, regular exercise is well known to trigger the release of serotonin and dopamine, which neurotransmitters help to overcome the symptoms of depression and anxiety [48].

### 2.5. Antioxidant and Anti-Inflammatory Effects of Exercise

A number of studies have shown that besides many favorable effects on different organ system functions (e.g., cardiovascular or metabolic), physical exercise is able to decrease proinflammatory markers and improve antioxidant status systemically [49–51]. Regular exercise mitigates reactive oxygen species-mediated cell damage by boosting antioxidant functions and reducing C-reactive protein, interleukin-6, and tumor necrosis factor-alpha (TNF-*α*) levels [52, 53]. Furthermore, it is proved to reduce reactive oxygen species (ROS) production; therefore, it plays a key role in the maintenance of redox balance [54]. Regular training leads to the adaptation of the antioxidant capacity and protects the cells against adverse oxidative processes [55]. Sustained exercise has been demonstrated to be essential not only in the elimination of oxidative stress but also in the prevention of the abovementioned complex disorders, such as type II diabetes, metabolic syndrome, and cardiovascular and even neurodegenerative diseases [56]. Numerous animal studies also demonstrated the antioxidant effects of physical exercise by the enhancement of several enzymatic pathways including glutathione (GSH) and the heme oxygenase (HO) system [57, 58]. Szabo et al. supported that 12 weeks of sustained training is an efficient method to enhance the antioxidant GSH and nitrotyrosine-3 levels as well [59]. Moreover, in a hormone-depleted rat model, 6 weeks of physical exercise was proved to be a key process in the amelioration of antioxidant status by enhancing the HO enzyme system [31]. Besides GSH and HO, superoxide dismutase (SOD) is also considered to be a first-line defense participant against oxidative stress. According to animal studies, a significant elevation can be observed in the production of SOD as a result of recreational training [60, 61]. Proinflammatory markers (e.g., myeloperoxidase (MPO), TNF-*α*, and IL-6) are the main contributors of ROS generation and consequently oxidative stress. Exercise, however, has also been shown to be effective in the reduction of these well-known inflammatory factors. Several studies showed that increased physical activity resulted in diminishing inflammatory processes [49, 62, 63]. It is clarified that 6-week-long voluntary exercise is effective enough to reduce MPO activity and the level of TNF-*α* in hormone-depleted rats [64]. It can therefore be concluded that exercise represents a potent anti-inflammatory and antioxidant strategy in healthy individuals and under pathological conditions as well. It participates in reducing the risk of morbidity and mortality through its direct and positive impacts on human health.

### 2.6. Cancer Prevention

Studies provide numerous evidence that physical activity reduces the risk of at least a dozen cancer types, including breast, colon, prostate, or lung cancer [65]. In different chemical-induced or genetic tumor models, all of the training modalities mentioned in this review have been shown to be effective in reducing tumor growth or metastasis [66, 67]. However, the underlying mechanisms of this wide-ranging protection are not yet totally clarified, but the possible mediators are inflammation-, antioxidant-, and immune cell-related [68]. During physical activity, a significant increase in muscle-derived myokines and intensified mobilization of immune cells can be observed in the plasma. While myokines have antiproliferative effects, immune cells can be the most powerful components in the fight against cancer [69, 70]. Pedersen et al. found a marked decrease in tumor incidence and growth as a result of voluntary wheel running in 5 different tumor types. They clarified that natural killer cells have a predominant role in this type of control of tumor growth; with the induction of stimulatory cytokines, enhancement of NK cell-related activated receptors, and their intensified mobilization, they have a major role in the training-related control of tumor growth [71]. Moreira et al. revealed that even a short-term voluntary exercise decreased tumor growth and metastatic processes in a tumor-bearing rat model [72]. A promising observation was made by Hagar et al. as well that 8 weeks of training enhanced antitumor immune processes, thus suppressing tumor growth in mice [73]. Furthermore, aerobic exercise resulted in enhanced tumor cell apoptosis, decreased tumor weight, and diminished cell proliferation in a tumor-bearing rat model compared to sedentary animals [74]. Physical activity has an unquestionable role in the primary prevention of cancerous processes; however, it is also extremely important in terms of health promotion after the diagnosis. With the help of exercise, aerobic capacity and muscle strength increase, while disease-free survival may extend [75].

## 3. Characteristics of Animal Exercise Protocols

**Figure 1:**
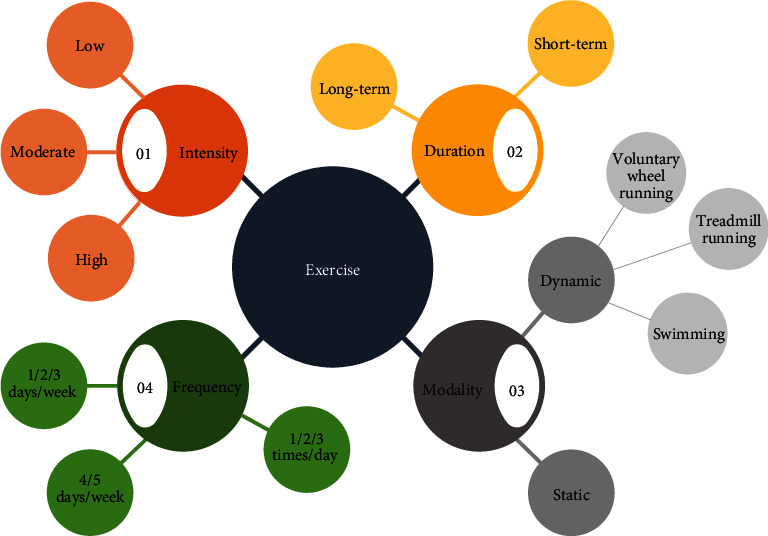
Fundamental elements of the exercise protocol. Intensity, duration, frequency, and modality are the four key components of an exercise protocol. Further variants of these subgroups can be used to refine the form of training.

Animal models are essential in basic research including every research field; thereby, choosing a well-designed exercise protocol for the appropriate experiments is fundamental. Before initiating any exercise study, the most important step is the proper selection of the animal model, as the objectives of exercise-related research studies may be different. For exercise training, rodents (rats and mice) are the most commonly used animals due to their many advantages. Rodents are the most affordable species for animal studies, thanks to their low breeding cost. They have high fertility and a relatively short gestational period with many offspring. Another advantage of using rodents is that they require comparatively small living space, and the experimental apparatus designed for mice or rats are also easily accessible [76]. Additionally, the capability of choosing genetically modified strains designed for specific diseases has also popularized these species in every research field. Despite the several advantages of rodents in animal research, few limitations are present in their application. In most animal studies, including exercise research, gender differences may interfere with the results and make it difficult to generalize data for both sexes, and although human and rodent genes are largely similar, the small but more important differences in the details (e.g., receptors) make rodents unsuitable for some research areas. Considering all aspects, after choosing the applicable animal model, we must consider the elementary factors of exercise. Physical activity can be characterized according to its intensity, duration, modality, and frequency [7, 77] (Figure 1).

Within the intensity of the training, we can distinguish between the low- and high-intensity physical activities; in terms of modality, we can differentiate between dynamic training and static training. According to the duration, exercise can be divided into short- or long-term exercise, while the frequency of training can also be further subdivided into several groups according to the goal of the study [8]. Exercise must meet different criteria according to the purpose of the research. As exercise research studies are designed for assessing the impact of physical activity on several organ systems, it is crucial to optimize the exercise protocol according to the goal of the study. Furthermore, for the successful outcome, exercise training must consist of several fundamental phases including regeneration time. At the beginning of the study (Phase I), animals should be familiarized with the applied training in order to prevent any injuries or exercise-induced stress. In this phase, adaptation to the environment as well as acquaintance with new forms of movement (e.g., swimming or running) takes place. Then, the planned training with the appropriate intensity, modality, and duration is performed (Phase II). Last but not least, in the case of a daily exercise period, resting time is also necessary for the animals (Phase III) in order to restore physical energy after training [8]. These previously described factors fundamentally determine the outcome of the experiment; thus, their understanding and accurate application are essential for adaptation to human physiology. In the following, we summarize the most often used aerobic and anaerobic training models with their possible areas of application.

## 4. Aerobic Exercise Models

The most commonly used aerobic exercise models in different research fields are voluntary wheel running, forced wheel running, swimming, and treadmill running (Table 1). The aim of these studies can be twofold: to determine the role of sport in disease prevention or to allocate its wide-ranging effects on preexisting disorders.

### 4.1. Wheel Running

Voluntary wheel running is a form of exercise where animals have free access to a metal wheel for the whole training time. The running wheel is usually built into the cages of the animals; therefore, they can use the apparatus according to their needs at a lower intensity, any time of the day for any length of time [76]. Running wheels are suitable for smaller rodents (e.g., mice or rats) and are nowadays often equipped with an activity tracking device, which allows the scientist to track down the running distance of the animals. This recreational training is the most stress-free modality of exercise; thus, it is suitable for aging studies and also in conditions where it is important to avoid strenuous exercise [30]. As for the duration of the experiment, voluntary wheel running is applicable for short- and long-term interventions as well. Due to its voluntary, nonstrenuous nature, it is often used for cardiovascular and metabolic studies, but it is suitable for almost every research area [78–80]. Long-term voluntary wheel running is considered to be protective against cardiac injury [81] and an effective tool to enhance antioxidant mechanisms [82]. According to Cunha et al., 3-week-long wheel running improved overall antioxidant status in mice [83]. Additionally, 12 weeks of wheel running exercise exerted its positive influence on lipid metabolism by resulting in a significant decrease in the level of plasma triglyceride and leptin [21]. Along with metabolic effects, long-term voluntary wheel running favorably affected bone properties as well in young mice [84]. Voluntary wheel running was also a convenient exercise protocol in neurodegenerative disorders, as it effectively mitigated impaired spatial memory and neuropathological changes in aging rats through complex biochemical processes [85]. This type of aerobic exercise is also a popular therapeutic approach to tumor prevention and treatment in cancer research [86].

A very similar form of movement to voluntary wheel running is forced wheel running. Forced wheel running differs from the previously mentioned form of wheel running in that its wheel is centrally motorized. This automatically rotating wheel is connected to a specific software program, which allows the scientist to adjust training intensity throughout the running from low to intermediate levels. This training modality offers better control of exercise parameters compared to voluntary wheel running. Forced wheel running is suitable for short-term and long-term interventions as well, depending on the purpose of the study. Similar to voluntary wheel running, this type of exercise is applicable in many areas of research.

### 4.2. Treadmill Running

Treadmill running is considered to be a forced training model, usually applied with smaller rodents or dogs. Unlike voluntary wheel running, during this exercise, animals are removed from their cages and forced to run on a treadmill. Scientists can change several parameters according to the goal of the study; it allows them to perform moderate- or high-intensity training by adjusting speed, duration, or inclination [76]. Treadmill running is a widely used exercise modality, especially in cardiovascular or metabolic research studies. It has been proved that high-intensity treadmill training is an efficient method to reduce cardiovascular risk factors. Haram et al. confirmed that it was able to decrease blood pressure and improve endothelial function and different metabolic parameters as well [87]. It was also reported that high-intensity exercise stimulated mitochondrial biogenesis, thereby contributing to cardiac improvements [88]. Furthermore, this kind of exercise is an applicable method to recreate exercise-induced physiological cardiac hypertrophy. Kemi et al. proved that 4 weeks of intensity-controlled treadmill running caused elevated ventricular weights and normalized the structure and function of the heart in female and male mice [89]. The work of Kim and Hwang discussed that a short-term (3 weeks) treadmill training was able to improve oxidative parameters in rats with cardiomyopathy [90], while the work of Cechetti et al. demonstrated that a moderate-intensity treadmill training mitigated oxidative damage in the rat hippocampus, therefore contributing to cognitive improvements [91]. According to the results of Wu et al., 9 weeks of treadmill exercise has beneficial effects against depression-like behavior in rats [92]. In addition, 3 weeks of forced running has also been shown to be beneficial in doxorubicin-induced liver disease through the normalization of oxidative stress markers [93]. Treadmill running is an often-applied exercise modality in cardiovascular therapy-connected research studies; however, conditions occurring during this kind of exercise are generally stressful, which circumstances may interfere with the experimental results, and for that reason, it is not recommended in aging studies. In addition to these areas of use, this type of training at different intensities is able to change the microstructure of the bones, according to Liu et al. [94]. Treadmill running is effective in a hormone-depleted female rat model as well, as its long-term application significantly increased bone mass and strength in young and adult rats [95].

### 4.3. Swimming

Similar to treadmill running, swimming is also a forced training model. This exercise modality obviously requires a simple swimming apparatus (e.g., a tank), which has to be large enough for the training. It is filled with 30-32°C water, the depth of which must be appropriate to the size of the animal [76]. In order to minimize the water-induced stress response, animals must be familiarized with the environment before the experiment. Unlike in the case of running exercises, here, sedentary control animals should also be placed in shallow water in order to exclude the stressful effects of water [96]. In this type of exercise, both the duration and the frequency can be adjusted according to the purpose of the experiment. Based on these factors, we can distinguish between the moderate- and strenuous-intensity exercises. Moderate training means 1 hour/day, 5 days/week for 8 weeks, while strenuous exercise requires an increasing duration of the sessions, finally reaching a 2.5-hour-long training period/day for also 8 weeks [97]. Furthermore, the swimming procedure can be used as an aerobic exercise with or without an attached weight workload [98]. Swimming with extra weight allows us to study the cardiovascular effects of an exhaustive, strenuous exercise. Olah et al. proved that a 3-hour-long swimming exercise with an extra 5% body weight attached to the animal resulted in elevated plasma troponin T and creatine kinase. Furthermore, they demonstrated that this kind of exhaustive training caused elevated apoptotic signaling and matrix metalloprotease dysregulation in the heart [99].

Moderate-intensity swimming, however, is a suitable exercise protocol to study physiological hypertrophy, similar to treadmill running. The findings of Evangelista et al. demonstrated that 90 min of swimming twice a day, 5 days a week for a 4-week-long period, induced physiological hypertrophy in mice and contributed to normalized heart function [100]. Besides cardiovascular effects, moderate-intensity swimming was proved to be efficient in complex metabolic mechanisms. Moustafa and Arisha clarified the beneficial changes of swimming exercise in terms of metabolic alterations [101]. Short-term swimming could be effective by decreasing blood glucose levels and improving insulin-connected pathways in diabetic rats [102]. Besides metabolic influence, swimming exerts anti-inflammatory effects by reducing proinflammatory cytokines in diabetic rats according to de Lemos et al. [103]. It has also been proven that 8 weeks of swimming training successfully mitigated the oxidative damage of the brain and increased its antioxidant status as well [104]. Similarly, Stone et al. found that moderate-intensity swimming was able to upregulate the expression of GSH, SOD, and catalase enzymes, thus ameliorating the antioxidant properties of the hippocampus [105]. According to the latest findings of Alomari et al., short-term swimming resulted in a significant improvement in short- and long-term memories in rats [106]. In this context, Park et al. proved that swimming ameliorated memory defects and psychological disorders by increasing serotonin expression and neurogenesis [107]. The areas of application of swimming extend to the osteoskeletal system as well, as clear results were obtained by Hart et al., who proved that 12-week-long swimming training was effective in the improvement of bone density, structure, and formation in a hormone-depleted female rat model [108].

## 5. Resistance (Anaerobic) Training

Resistance training is an exercise modality designed to enhance muscular strength, power, or physical capacity. In this type of training, external assistance (e.g., electric stimuli, surgery, and specific equipment) is essential to provoke the animals to perform the exercise. Resistance training is usually used for studies in connection with cognitive function and muscle hypertrophy or atrophy [11, 109].

### 5.1. Ladder Climbing

In this type of resistance training, rats are trained to climb a ladder with a load apparatus stabilized to their tail. To perform ladder climbing, no noxious stimuli or motivators are necessarily needed; thus, it can be considered a voluntary exercise. Animals need to be progressively familiarized and trained with climbing the specially designed ladder before the experiment. The intensity of this exercise is defined by how many climbing repetitions are performed during one training phase. It can be applied as a short-term or long-term exercise model as well. Due to the increased muscle workload, in this type of exercise modality, significant muscle hypertrophy is obtained [110]. Jung et al. clarified that 8 weeks of ladder climbing upregulated the muscle hypertrophy-related myokines in young and adult rats [111]. In addition to muscle hypertrophy, ladder models are increasingly used for studies involving the central nervous system. In this context, according to the results of Cassilhas et al., 8 weeks of ladder climbing exercise improved hippocampus-dependent memory tasks in rats [10]. It also increases cell proliferation and the expression of antiapoptotic proteins in the hippocampus [112].

### 5.2. Weight Lifting

Unlike humans, rodents can perform weight lifting by standing upright and lengthening their hindlimbs. In a specific squat training apparatus, additional weight is added to the animals by using a belt or a shoulder harness. The main disadvantage of this type of resistance training is the use of harmful stimuli in order to motivate the animals to complete the training. This protocol results in nearly 20% hypertrophy of the leg muscles; thus, it is suitable for research on muscle development [113].

### 5.3. Electric Stimulation of the Muscles

In order to perform this protocol, animals must be anesthetized. This model also requires an implanted electric stimulator placed into the muscle to be examined [114]. Scientists can control the degree of electric stimulation, which can occur bilaterally or unilaterally. This modality of training evokes significant muscle hypertrophy [115]. The advantage of this protocol is that cooperation of the animals is less needed as a result of anesthesia, although this also implies its disadvantage, as anesthetics may influence the physiology of the animal [76].

## 6. Concluding Remarks

All things considered, the positive effects of physical activity on overall health are unquestionable. Even though animal exercise models have their own limitations, the data obtained through their applications could bring us closer to solving global health issues. As seen, even short-term training can upregulate the antioxidant defense system and induce multifaceted beneficial effects throughout the body. With this nonpharmacological, health-promoting tool, a large percentage of noncommunicable diseases, including metabolic syndrome, CVDs, or even cancer, could be averted. In order to gain a better insight into exercise physiology and its impacts on health status, well-designed animal models are needed.

## Figures and Tables

**Table 1:** Detailed summary of different exercise modalities (advantages, disadvantages, and areas of application).

Type of exercise	Most common research areas	Advantage	Disadvantage
*Aerobic*			
Voluntary wheel running	Aging, cardiovascular research, behavioral research, cancer research, metabolic research, stroke, liver and kidney disease, bone and muscle physiology, memory	Nonstressful	Uncontrollable (intensity, duration) Possible paw injuries
Forced wheel running	Aging, cardiovascular research, behavioral research, cancer research, metabolic research, stroke, liver and kidney disease, bone and muscle physiology, memory	Controllable (intensity, duration, frequency)	Stressful Possible paw injuries
Treadmill running	Cardiovascular research, behavioral research, cancer, metabolic research, stroke, liver and kidney disease, bone and muscle physiology, memory	Controllable (intensity, duration, frequency)	Stressful Possible paw injuries Expensive apparatus
Swimming	Aging, cardiovascular research, behavioral research, cancer, metabolic research, stroke, liver and kidney disease, bone and muscle physiology, memory, spinal cord injury	No paw injuries Less expensive apparatus	Stressful
*Anaerobic (resistance)*			
Ladder climbing	Memory, behavioral research Muscle hypertrophy model	With familiarization, it is less stressful	Long familiarization process
Weight lifting	Muscle hypertrophy model	Similar to human training Quantitative	Stressful to animals Special equipment is needed
Electric stimulation of the muscles	Muscle hypertrophy model Muscle injury	Controlled muscle stimulation Quantitative	Anesthesia Surgery Artificial muscle contraction
